# Integrative analysis of KIF4A, 9, 18A, and 23 and their clinical significance in low-grade glioma and glioblastoma

**DOI:** 10.1038/s41598-018-37622-3

**Published:** 2019-03-14

**Authors:** Sang Yeon Cho, Sungha Kim, Gwanghun Kim, Parul Singh, Dong Woon Kim

**Affiliations:** 1 Department of Anatomy, Brain Research Institute, Chungnam National University School of Medicine, Daejeon, 35015 Republic of Korea; 2 Department of Clinical Research, Korea Institute of Oriental Medicine, Daejeon, 34054 Republic of Korea; 3 Department of Biomedical Sciences, Seoul National University College of Medicine, Seoul, 03080 Republic of Korea; 4 Department of Microbiology and Immunology, Chonbuk National University School of Medicine, Jeonju, 54907 Republic of Korea; 5 Department of Medical Science, Chungnam National University School of Medicine, Daejeon, 35015 Republic of Korea

## Abstract

To determine the prognostic significance of kinesin superfamily gene (KIF) expression in patients with brain cancer, including low-grade glioma (LGG) and glioblastoma (GBM), we comprehensively analyzed KIFs in 515 LGG and 595 GBM patients. Among KIFs, KIF4A, 9, 18A, and 23 showed significant clinical implications in both LGG and GBM. The mRNA and protein expression levels of KIF4A, 9, 18A, and 23 were significantly increased in LGG and GBM compared with those in the normal control groups. The mRNA expression levels of KIF4A, 9, 18A, and 23 in LGG were significantly increased in the high-histologic-grade group compared with those with a low histologic grade. Genomic analysis showed that the percent of mRNA upregulation of KIF4A, 9, 18A, and 23 was higher than that of other gene alterations, including gene amplification, deep deletion, and missense mutation. In addition, LGG patients with KIF4A, 18A, and 23 gene alterations were significantly associated with a poor prognosis. In survival analysis, the group with high expression of KIF4A, 9, 18A, and 23 mRNA was significantly associated with a poor prognosis in both LGG and GBM patients. Gene Set Enrichment Analysis (GSEA) revealed that high mRNA expression of KIF4A, 18A, and 23 in LGG and GBM patients showed significant positive correlations with the cell cycle, E2F targets, G_2_M checkpoint, Myc target, and mitotic spindle. By contrast, high mRNA expression of KIF9 in both LGG and GBM patients was significantly negatively correlated with the cell cycle, G_2_M checkpoint, and mitotic spindle pathway. However, it was significantly positively correlated with EMT and angiogenesis. This study has extended our knowledge of KIF4A, 9, 18A, and 23 in LGG and GBM and shed light on their clinical relevance, which should help to improve the treatment and prognosis of LGG and GBM.

## Introduction

Glioblastoma (GBM) accounts for 60–70% of all gliomas and remains one of the most challenging malignancies worldwide^1^. The characteristics of GBM, disseminating within the brain, severely limit the efficacy of surgery and radiotherapy^2^. Low-grade gliomas (LGGs) constitute grade I and grade II tumors of the astrocytic lineage and grade II tumors of the oligodendroglial lineage. Although LGGs are typically slow-growing, they may be associated with significant morbidity and mortality due to recurrence and malignant progression, even in the setting of optimal resection^3^. Secondary glioblastomas can also progress from low-grade diffuse astrocytoma or anaplastic astrocytoma^4^. Each of these features has demanded the identification of new targets for GBM and LGG for gene/antibody therapy. In both GBM and LGG, features of cellular physiology such as mitosis and cell motility are important new targets. Because the cell cycle is a conserved process necessary for cell growth and development, cell cycle aberrations are a hallmark of cancer^5^. Accordingly, there is a need to identify therapeutic targets capable of regulating the cell cycle for both GBM and LGG.

The kinesin superfamily genes (KIFs) play important roles related to the cell cycle. They have been shown to participate in chromosomal and spindle movements during mitosis and meiosis. KIFs also transport organelles, protein complexes, and mRNAs to specific destinations in a microtubule- and ATP-dependent manner^6^. Increasing evidence has indicated that kinesin proteins play critical roles in the genesis and development of human cancers^7^. Several KIF proteins show aberrant overexpression in various cancer cells^7^. KIF4A overexpression has a strong association with the poor prognosis of non-small cell lung cancer^8^. KIF11 plays a driver of invasion, proliferation, and self-renewal in glioblastoma^2^. Increased expression of KIF20A indicates poor prognosis of glioma patients^9^. KIF20B is strongly overexpressed in bladder cancer tissues, and the downregulation of endogenous KIF20B leads to cytokinesis defects^7^. KIF14 expression in gliomas is tumor-specific and is increased in more aggressive tumors^10^. However, to our knowledge, insufficient studies have investigated the correlation between KIFs and LGG or GBM. Previous studies have shown that most mitotic kinesins, which are involved in cell division, are associated with tumor progression. Some non-mitotic kinesins, which are principally involved in intracellular transport, were also identified in tumorigenesis^11^. Here, we aimed to determine the prognostic significance of KIF expression in patients with LGG and GBM using TCGA data bioinformatically.

## Results

### mRNA and protein expression of KIF4A, 9, 18A, and 23 in LGG and GBM

**Figure 1:**
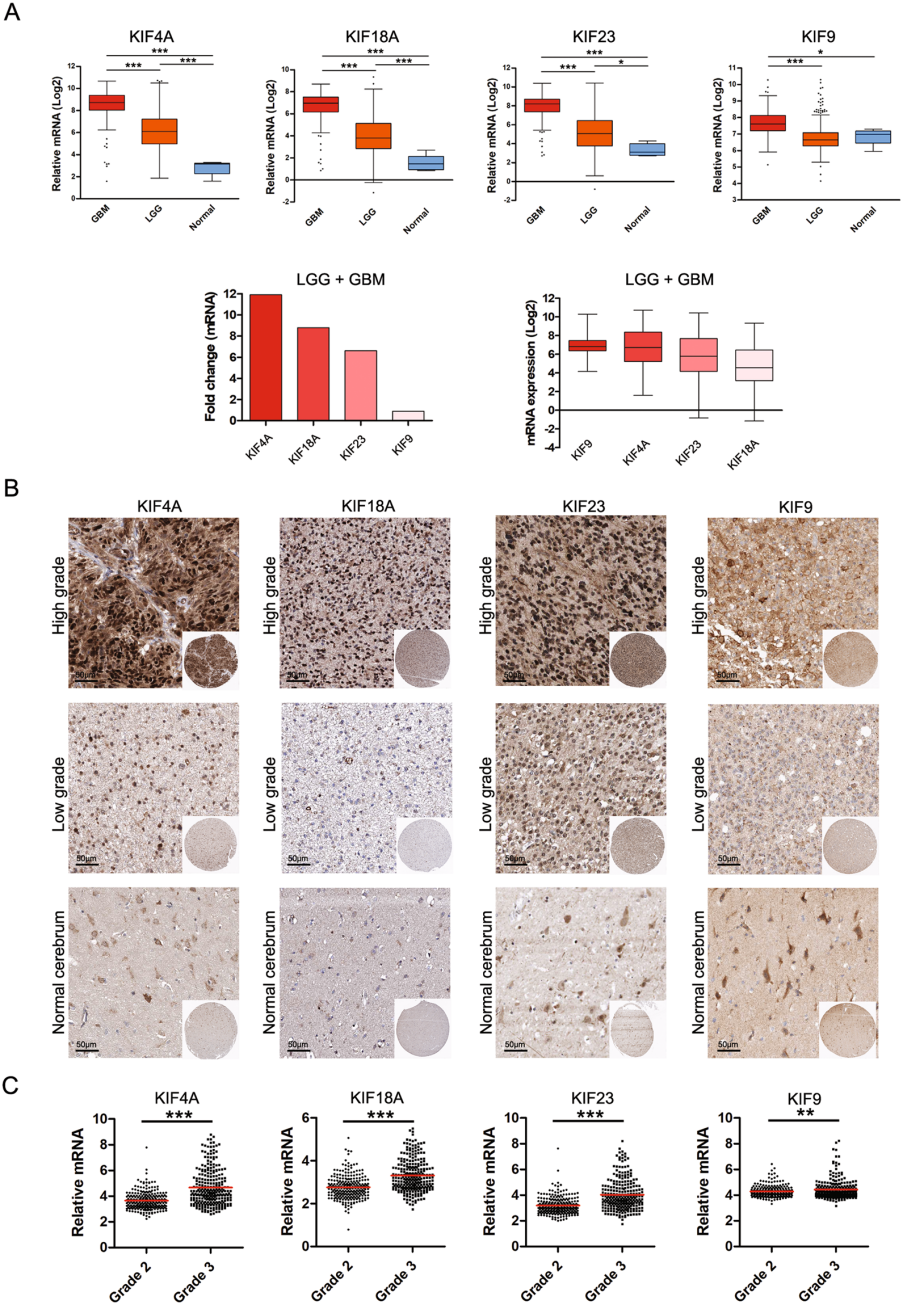
mRNA and protein expression of KIF4A, 9, 18A, and 23 in LGG and GBM. (**A**) The relative mRNA expression of KIF4A, 9, 18A, and 23 was compared among the normal control, LGG, and GBM (upper). The mRNA expression of KIF4A, 9, 18A, and 23 in LGG and GBM merged dataset was compared with that in normal samples in the TCGA dataset (lower). The fold change values of KIF4A, 9, 18A, and 23 were 11.9, 0.9, 8.8, and 6.6, respectively (lower left). The relative mRNA expression of KIF4A, 9, 18A, and 23 was compared in LGG and GBM merged dataset (lower right). (**B**) Representative IHC images of KIF4A, 9, 18A, and 23 in normal cerebrum, low-grade glioma, and high-grade glioma in Human Protein Atlas. Image available from v18.proteinatlas.org^18–20^. The scale bar indicates 50 µm. (**C**) Comparison of the mRNA expression of KIF4A, 9, 18A, and 23 in LGG between histologic grades 2 and 3. The distribution was compared between the two groups by *t*-test. A p-value < 0.05 was deemed to indicate statistical significance. *p < 0.05, **p < 0.01, ***p < 0.001.

To investigate KIF genes affecting the progression of LGG and GBM and the prognosis of the patients, we investigated genes which are significantly increased in LGG and GBM than in the normal group (Supplementary Figs 1 and 2). Then we discovered four increased genes, KIF4A, 9, 18A, and 23, which were significantly associated with poor prognosis in LGG and GBM patients. The kinesin superfamily proteins (KIFs) including KIF4A, 9, 18A and 23 are ATP dependent microtubule-based motor proteins. Four of the KIF genes were not identical in function in the cell, but all of the previous studies showed a strong association with the formation and arrangement of chromosomes or cytokinesis^12–17^. When the expression levels of the KIF4A, 9, 18A, and 23 genes were compared among GBM, LGG and normal group, the levels of KIF4A, 18A and 23 showed a significant progressive increase with GBM and LGG from normal group (Fig. 1A upper panel). When the mRNA expression of KIF4A, 9, 18A, and 23 in the LGG and GBM merged dataset was compared with that in normal groups, KIF4A, 18A, 23, and 9 exhibited 11.9-, 8.8-, 6.6, and 0.9-fold change compared to the normal group respectively (Fig. 1A lower left panel and Table 1). Interestingly, however, KIF9 showed the highest mRNA expression in four the KIF genes, even though KIF9 was not significantly increased in LGG than in normal group (Fig. 1A lower right panel).The protein expression of KIF4A, 9, 18A, and 23 among control, low-grade glioma, and high-grade glioma was also examined using The Human Protein Atlas (HPA) portal^18–20^. KIF4A, 9, 18A, and 23 genes showed that, as the degree of glioma increased, the degree of staining also increased (Fig. 1B). In addition, the protein expression and localization of four the KIF genes were identified in glial cells in normal group and the glioblastoma cell line, and glioma (Supplementary Figs 3–6). Although the four KIFs in normal glial cells, the glioblastoma cell line, and glioma tissues showed internal discrepancies to identify the targets as nuclear or cytoplasmic especially between the immunofluorescence and immunohistochemistry, KIF4A, 18A, and 23 were able to be identified in nuclear localization, however, KIF9 was only localized in cytoplasm. In the histologic grade 3 group, the mRNA expression was significantly increased compared with that in the grade 2 group (Fig. 1C). In addition, the mRNA expression of KIF4A, 9, 18A, and 23 was significantly increased in astrocytoma compared with that in oligoastrocytoma and oligodendroglioma (Supplementary Fig. 7A). When the mRNA expression was examined in terms of laterality and location of the brain lobe, no significant differences in expression were identified (Supplementary Fig. 7B,C).

**Table 1:** KIF family genes of GBM and LGG.

Symbol	Gene name	Chromosome location	Fold change (Log)	Alteration (%)
KIF4A	Kinesin Family Member 4A	Xq13.1	11.9	1.1
KIF9	Kinesin Family Member 9	3p21.31	0.9	1.0
KIF18A	Kinesin Family Member 18A	11p14.1	8.8	0.5
KIF23	Kinesin Family Member 23	15q23	6.6	0.3

### Genomic analysis of KIF4A, 9, 18A, and 23 in LGG and GBM

**Figure 2:**
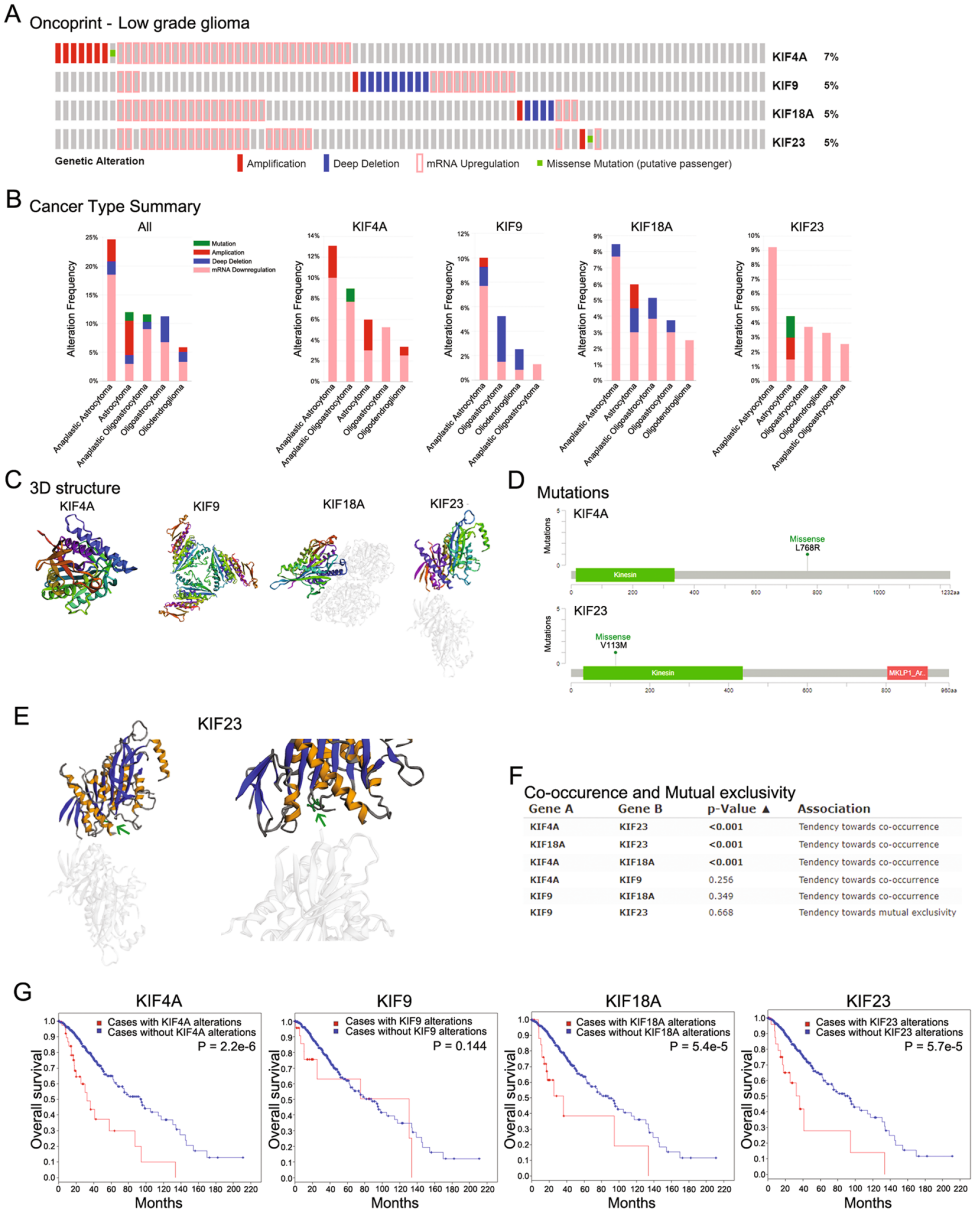
Genomic analysis of KIF4A, 9, 18A, and 23 in LGG. (**A**) The oncoprints of KIF4A, 9 18A, and 23 were identified. Genetic alterations of KIF4A, 9, 18A, and 23. The column represents LGG patients, and the row represents gene alterations, including amplification, deep deletion, mRNA upregulation, and missense mutation. (**B**) Genetic alterations summarized according to cancer type of LGG. (**C**) The 3D crystal structure of KIF4A, 9, 18AA, and 23 is shown in rainbow colors (from N- to C- terminal ends). (**D**) The mutations of KIF4A and 23 were plotted. The kinesin motor domain is displayed in green with 15–336 amino acids. In the 768 amino acid sequence of KIF4A, Leucine was changed to Arginine. In the 133 amino acid sequence of KIF23, Valine was changed to Methionine. (**E**) The 3D structure of KIF23 implemented with missense mutations was plotted. In the kinesin motor domain of KIF23, the mutated part was marked with a green arrow. (**F**) Co-occurrence and mutual exclusivity were examined with KIF4A, 9, 18A, and 23. (**G**) Kaplan–Meier survival analysis of KIF4A, 9, 18A, and 23 was conducted with and without gene alterations. The p-value for Kaplan–Meier curves was calculated using the log-rank test.

Genomic analysis of KIF4A, 9, 18A, and 23 was conducted using cBioportal (http://www.cbioportal.org) (Fig. 2)^21,22^. Genetic alteration of KIFs was investigated in LGG (Supplementary Fig. 8A). Specifically, genetic alteration of KIF4A, 9, 18A, and 23 was analyzed and depicted as oncoprints representing amplification, deep deletion, mRNA upregulation, and missense mutation (Fig. 2A). Each genetic alteration of KIF4A, 9, 18A, and 23 was summarized between histologic types (Fig. 2B). The crystal 3D structure of kinesin motor domain in KIF4A, 9, 18A, and 23 was monomeric, homo trimer, hetero trimer and monomeric respectively. The 3D structures were presented rainbow colors (from N- to C- terminal ends) (Fig. 2C). In particular, missense mutation was identified in KIF4A and 23, but the mutation in kinesin motor domain was only verified in KIF23 (Fig. 2D). The specific crystal 3D structure modification of KIF23 is presented in Fig. 2E. Co-occurrence and mutual exclusivity were examined among KIF4A, 9, 18A, and 23. However, only the co-occurrence of KIF4A, 18A, and 23 was significantly identified, and there was no mutual exclusivity (Fig. 2F). Survival analysis of KIF4A, 9, 18A, and 23 with and without each gene alteration was conducted (Fig. 2G). LGG patients with KIF4A, 18A, and 23 gene alterations showed significantly poor overall survival compared with LGG patients without these gene alterations.

**Figure 3:**
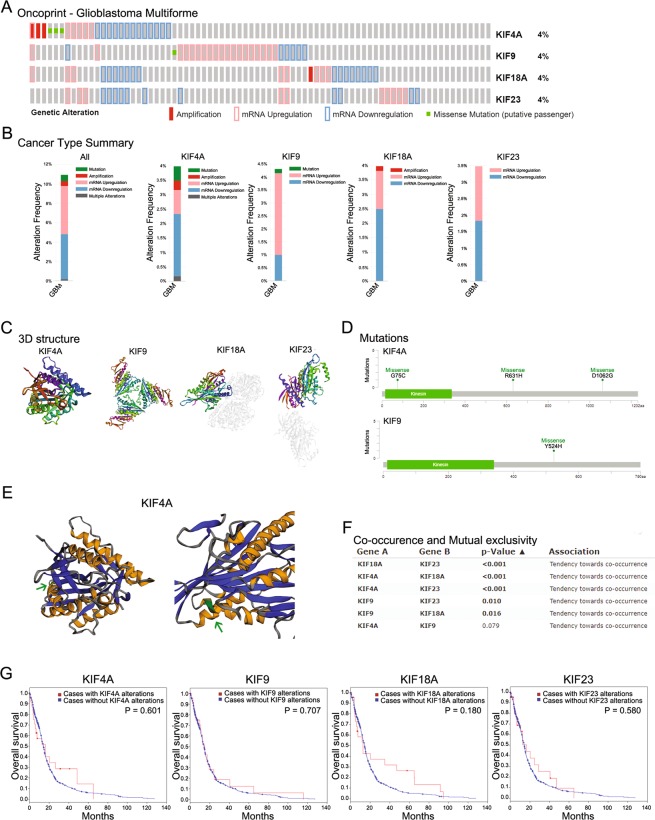
Genomic analysis of KIF4A, 9, 18A, and 23 in GBM. The oncoprints of KIF4A, 9 18A, and 23 were identified. Genetic alteration of KIF4A, 9, 18A, and 23. Column represents GBM patients, and row represents gene alteration including amplification, deep deletion, mRNA upregulation, and missense mutation. (**B**) Genetic alteration summarized according to cancer type of GBM. (**C**) The 3D crystal structure of KIF4A, 9, 18A, and 23 is shown in rainbow colors (from N- to C- terminal ends). (**D**) The mutations of KIF4A and 9 were plotted. The kinesin motor domain is displayed in green with 15–336 amino acids. In the 75 amino acid sequence of KIF4A, Glycine was changed to Cysteine. Arginine, the 631th amino acids, was changed to Histidine in KIF4A. Aspartate, the 1062th amino acid, was changed to Glycine in KIF4A. In KIF9, Tyrosine, the 524th amino acid, was changed to Histidine. (**E**) The 3D structure of KIF4A implemented with missense mutations was plotted. In the kinesin motor domain of KIF4A, the mutated part was marked with a green arrow. (**F**) Co-occurrence and mutual exclusivity were examined for KIF4A, 9, 18A, and 23. (**G**) Kaplan–Meier survival analysis of KIF4A, 9, 18A, and 23 was conducted with and without alterations in overall survival. The p-values for Kaplan–Meier curves were calculated using the log-rank test.

Genetic alteration of KIFs was also investigated in GBM (Supplementary Fig. 8B). KIF4A, 9, 18A, and 23 in GBM were analyzed and depicted as oncoprints representing amplification, deep deletion, mRNA upregulation, and missense mutation (Fig. 3A). Each genetic alteration of KIF4A, 9, 18A, and 23 in GBM was summarized (Fig. 3B). The crystal 3D structure of kinesin motor domain in KIF4A, 9, 18A, and 23 was monomeric, homo trimer, hetero trimer and monomeric respectively. The 3D structures were presented as rainbow colors (from N- to C- terminal ends) (Fig. 3C). Missense mutation was identified in KIF4A and 23, but the mutation in kinesin motor domain was only verified in KIF4A (Fig. 3D). The specific crystal 3D structure modification of KIF4A is presented in Fig. 3E. Co-occurrence and mutual exclusivity were examined among KIF4A, 9, 18A, and 23. However, only the co-occurrence of KIF4A, 18A, and 23 was significantly identified, and no mutual exclusivity was found (Fig. 3F). Survival analysis of GBM patients with and without KIF4A, 9, 18A, and 23 gene alterations was conducted; however, it showed no significant difference in survival (Fig. 3G).

### Survival effects of KIF4A, 9, 18A, and 23 on LGG and GBM patients

**Figure 4:**
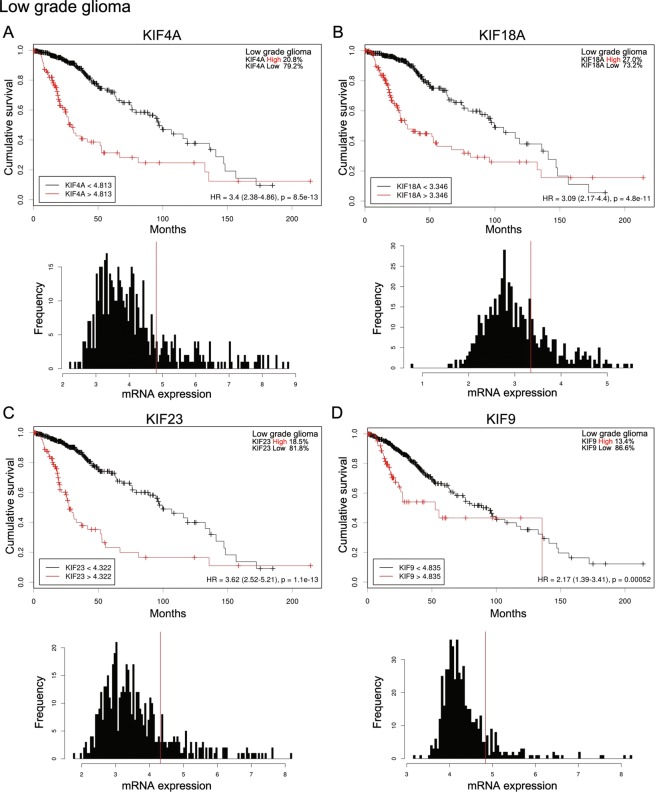
Effects of KIF4A, 9, 18A, and 23 on survival of LGG patients. The cutoff value determination in Cutoff Finder was based on survival, with significance based on the log-rank test for patient outcome (http://molpath.charite.de/cutoff/assign.jsp). (**A**) High mRNA expression of KIF4A was significantly associated with poor prognosis (hazard ratio [HR]: 3.4 [95% confidence interval (CI): 2.38–4.86]). In histograms of KIF4A mRNA expression, vertical red lines designate 4.813 as the optimal cutoffs of mRNA derived from the log-rank test. (**B**) High mRNA expression of KIF18A was significantly associated with: 3.09 [95% CI: 2.17–4.40]). In histograms of KIF18A mRNA expression, vertical red lines designate 3.346 as the optimal cutoffs of mRNA derived from the log-rank test. (**C**) High mRNA expression of KIF23 was significantly associated with poor prognosis (HR: 3.62 [95% CI: 2.52–5.21]). In histograms of KIF23 mRNA expression, vertical red lines designate 4.322 as the optimal cutoffs of mRNA derived from the log-rank test. (**D**) High mRNA expression of KIF9 was significantly associated with poor prognosis (HR: 2.17 [95% CI: 1.39–3.41]). In histograms of KIF9 mRNA expression, vertical red lines designate 4.835 as the optimal cutoffs of mRNA derived from the log-rank test.

**Figure 5:**
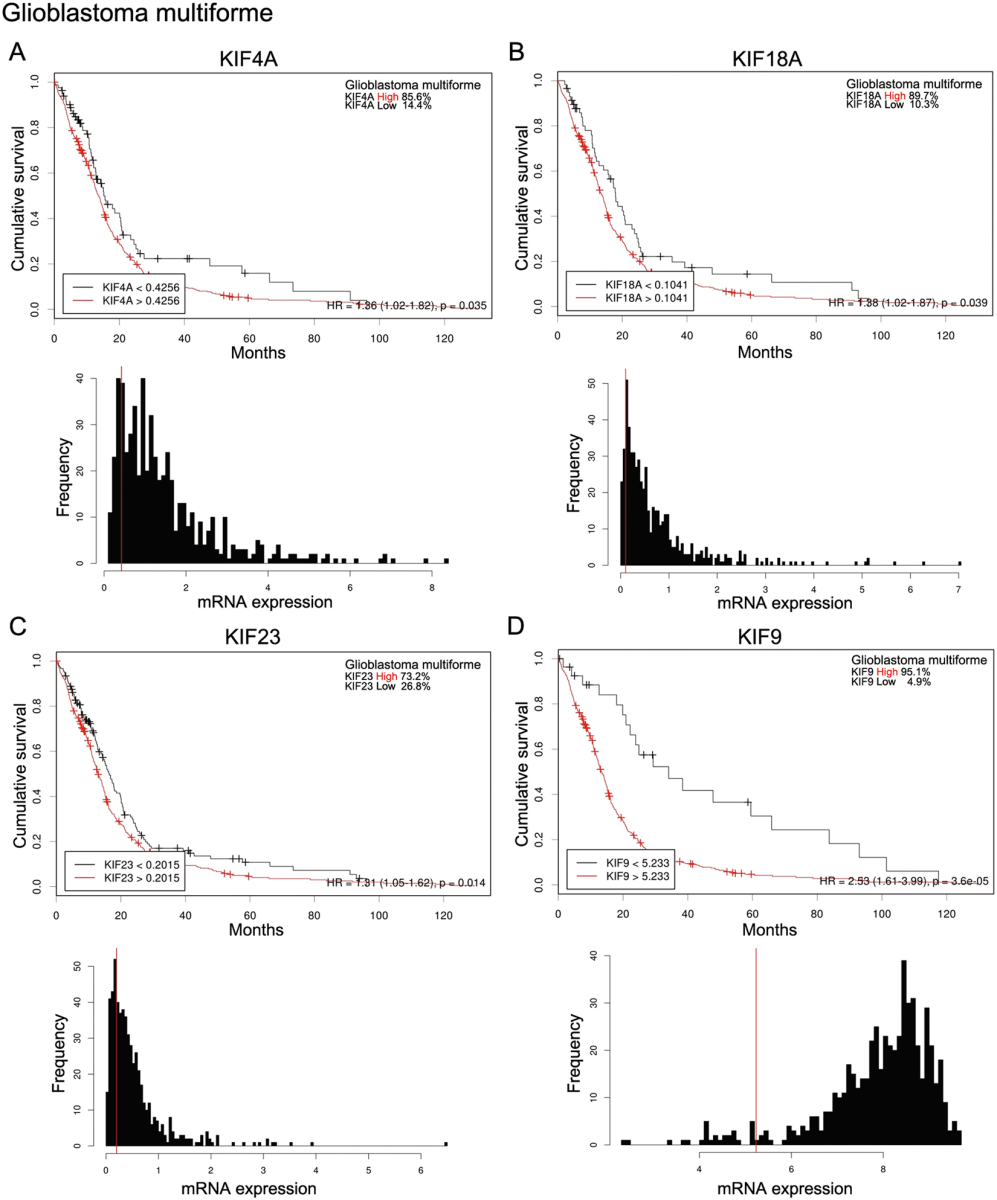
Effects of KIF4A, 9, 18A, and 23 on survival in GBM patients. The cutoff value determination in Cutoff Finder was based on survival, with significance based on the log-rank test for patient outcome (http://molpath.charite.de/cutoff/assign.jsp). (**A**) High mRNA expression of KIF4A was significantly associated with poor prognosis (HR: 1.36 [95% CI: 1.02–1.82]). In histograms of KIF9 mRNA expression, vertical red lines designate 0.4256 as the optimal cutoffs of mRNA derived from the log-rank test. (**B**) High mRNA expression of KIF18A was significantly associated with poor prognosis (HR: 1.38 [95% CI 1.02–1.87]). In histograms of KIF9 mRNA expression, vertical red lines designate 0.1041 as the optimal cutoffs of mRNA derived from the log-rank test. (**C**) High mRNA expression of KIF23 was significantly associated with poor prognosis (HR: 1.31 [95% CI: 1.05–1.62]). In histograms of KIF9 mRNA expression, vertical red lines designate 0.2015 as the optimal cutoffs of mRNA derived from the log-rank test. (**D**) High mRNA expression of KIF9 was significantly associated with poor prognosis (HR: 2.53 [95% CI: 1.61–3.99]). In histograms of KIF9 mRNA expression, vertical red lines designate 5.233 as the optimal cutoffs of mRNA derived from the log-rank test.

To determine the prognostic significance of the mRNA expression of KIF4A, 9, 18A, and 23 in LGG patients, we investigated the correlations between mRNA expression and overall survival in these patients (Fig. 4). Initially, Kaplan–Meier curves were used to plot overall survival against mRNA expression using Cutoff Finder (http://molpath.charite.de/cutoff). The cutoff value determination in Cutoff Finder was based on survival, with significance based on the log-rank test for patient outcome (http://molpath.charite.de/cutoff/assign.jsp). High levels of mRNA expression of KIF4A, 18A, 23, and 9 were significantly associated with a poor prognosis (KIF4A Hazard ratio [HR]: 3.4 [95% CI 2.38–4.86], KIF18A [HR]: 3.09 [95% CI 2.17–4.4], KIF23 [HR] 3.62 [95% CI 2.52–5.21], KIF9 [HR] 2.17 [95% CI 1.39–3.41]). To determine the prognostic significance of the mRNA expression of KIF4A, 9, 18A, and 23 in GBM patients, we investigated the correlations between the mRNA expression and overall survival of GBM patients (Fig. 5). Initially, Kaplan–Meier curves were used to plot overall survival with the mRNA expression using Cutoff Finder (http://molpath.charite.de/cutoff). High levels of mRNA expression of KIF4A, 18A, 23, and 9 were significantly associated with a poor prognosis (KIF4A Hazard ratio [HR]: 1.36 [95% CI 1.02–1.82], KIF18A [HR]: 1.38 [95% CI 1.02–1.87], KIF23 [HR] 1.31 [95% CI 1.05–1.62], KIF9 [HR] 2.53 [95% CI 1.61–3.99]).

### Analysis of networks and Gene Set Enrichment Analysis (GSEA) in LGG and GBM

**Figure 6:**
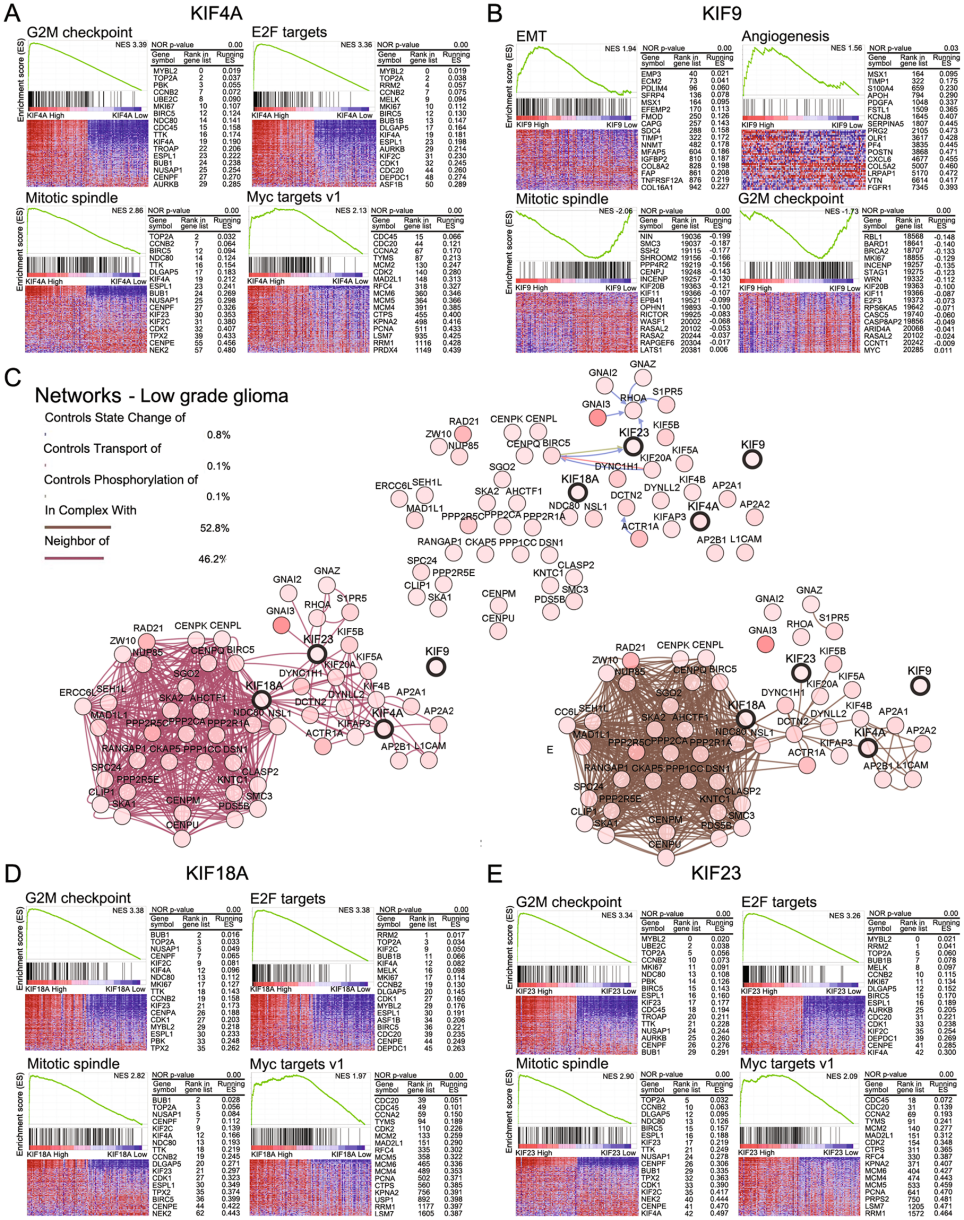
Network analysis and GSEA based on hallmark gene sets in LGG. The graph displays the enrichment gene set based on the pathway-provided hallmark genes in the top 10% vs. bottom 10% groups of KIF4A, 9, 18A, and 23 in LGG. (**A**,**D**,**E**) Pathways of the G_2_M checkpoint, E2F targets, mitotic spindle, and Myc targets v1 were increased in the high KIF4A, 18A, and 23 groups, which showed adverse effects on prognosis. (**B**) Pathways of EMT and angiogenesis were increased, and pathways of the mitotic spindle and G_2_M checkpoint were decreased in the high-KIF9 groups. (**C**) Network analysis of KIF4A, 9, 18A, and 23 in LGG was performed in cBioportal (http://www.cbioportal.org/)^21,22^. Each gene in the network is marked with a circle. Linkages related to state change, transport, phosphorylation, complex, neighbor among genes were connected by color lines.

**Figure 7:**
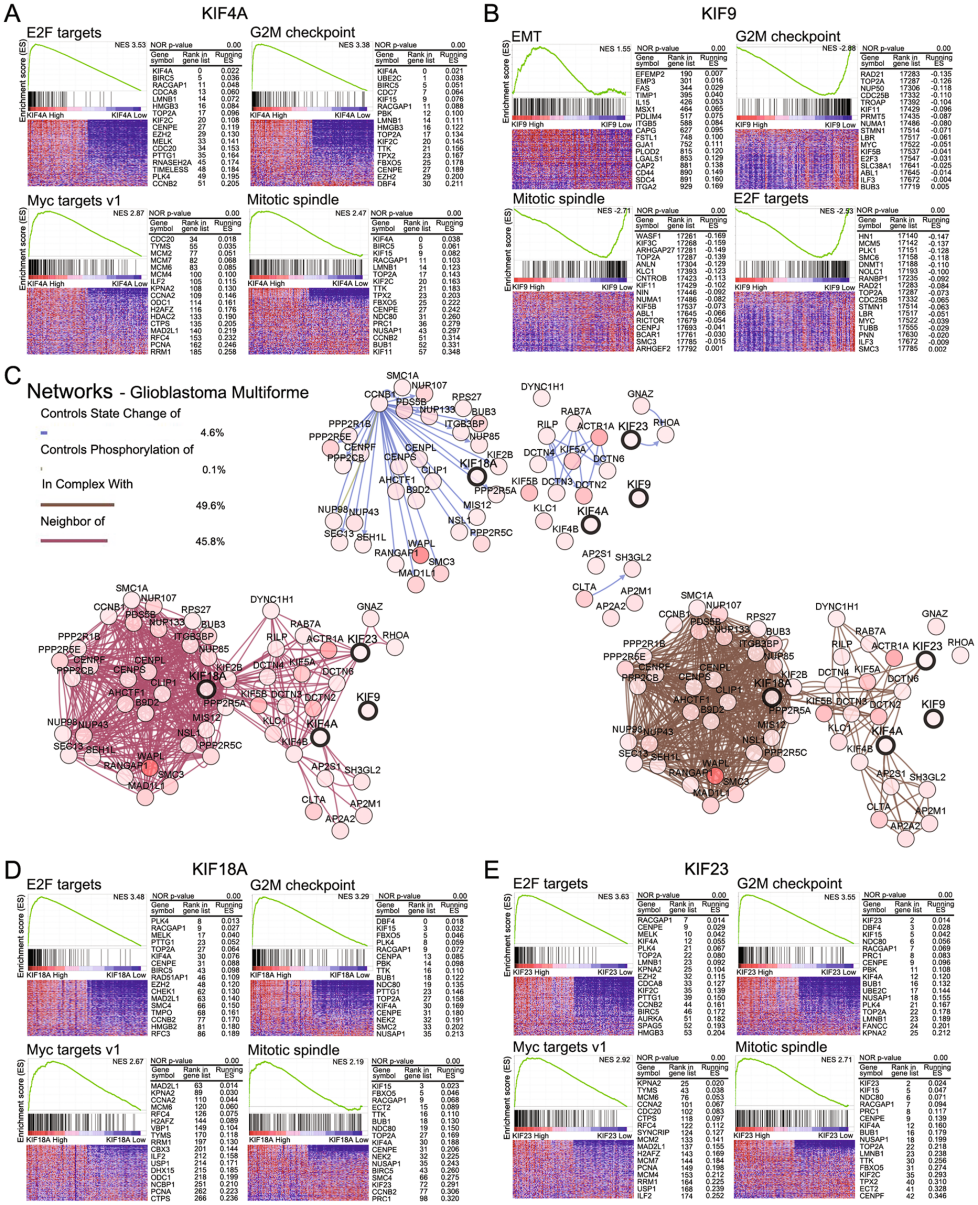
Network analysis and GSEA based on hallmark gene sets in GBM. The graph displays the enrichment gene set based on pathways provided by the hallmark database in the top 10% vs. bottom 10% of groups with KIF4A, 9, 18A, and 23 of GBM. (**A**,**D**,**E**) Pathways of E2F targets, G_2_M checkpoint, Myc targets v1, and mitotic spindle were increased in high KIF4A, 18A, and 23 groups, which had adverse effects on prognosis. (**B**) The EMT pathway was increased and pathways of the G_2_M checkpoint, mitotic spindle, and E2F targets were decreased in the high-KIF9 groups. (**C**) Network analysis of KIF4A, 9, 18A, and 23 in GBM was examined in cBioportal (http://www.cbioportal.org/)^21,22^. Each gene in the network is marked with a circle. Linkages related to state change, phosphorylation, complex, neighbor among genes were connected by color lines.

To examine the correlations with specific networks and increased pathways from GSEA, commonly increased or decreased pathways based on hallmark gene sets (“Hallmark gene sets summarize and represent specific well-defined biological states or processes and display coherent expression”, as defined by GSEA) were selected (Figs 6 and 7). Network analysis of KIF4A, 18A, 23, and 9 was performed using cBioportal (http://www.cbioportal.org/)^21,22^. In LGG patients, pathways of the G_2_M checkpoint, E2F targets, mitotic spindle, and Myc target v1 were increased in the high-expression KIF4A, 18A, and 23 groups and had a negative effect on prognosis (Fig. 6A,D,E). The pathways of EMT and angiogenesis were increased in the high-expression KIF9 group, with detrimental effects on prognosis. However, decreased pathways of the mitotic spindle and G_2_M checkpoint were identified (Fig. 6B). A comparable pattern was also found in GBM patients, and pathways of the E2F target, G_2_M checkpoint, Myc target v1, and mitotic spindle were also increased in the high-expression KIF4A, 18A, and 23 groups (Fig. 7A,D,E). However, increased pathway of EMT and decreased pathways of the G_2_M checkpoint, mitotic spindle, and E2F target were found in the high-expression KIF9 groups (Fig. 7B).

Enrichment map visualization of GSEA in LGG and GBM was performed to examine the effects of KIF4A, 18A, 23, and 9 on survival in LGG and GBM patients. GSEA was conducted between the high (top 10%) and low (bottom 10%) gene-expressing groups based on pathways provided by the Reactome database (Supplementary Fig. 9). KIF4A, 18A, and 23, which had detrimental effects on survival, had significant positive associations with the cell cycle and negative associations with GPCR ligand binding (Supplementary Fig. 9A–C) in LGG and GBM patients. On the other hand, KIF9, which also showed a detrimental effect on survival, had significant positive correlations with immune signaling (interferon, chemokine, PD1 signaling) and metabolic signaling (TCA cycle, acyl chain remodeling, bile acid metabolism, glucose metabolism) and negative correlations with transcription, mRNA metabolism, the neuronal system, NGF signaling, and the cell cycle (Supplementary Fig. 9D).

Chromosome circular plot analysis of KIF4A, 9, 18A, and 23 in LGG and GBM patients was performed to investigate which genes are associated with LGG and GBM via the Regulome Explorer (http://explorer.cancerregulome.org/) (Supplementary Fig. 10). The associations among KIF4A, 9, 18A, and 23 and other genes are depicted as colored lines at sites of the 23 chromosomes in LGG and GBM (Supplementary Fig. 10A–D). Specific gene networks with KIF4A, 9, 18A, and 23 were established, and the combined gene network for KIF4A, 9, 18A, and 23 was depicted and the specific genes of cell division and the spindle related were represented as black color and notch signaling related were represented as gray color (Supplementary Fig. 11).

## Discussion

The current standard care in GBM is surgical resection, followed by temozolomide chemotherapy and radiation, but GBM inevitably recurs due to a high rate of malignancy and intrinsic or rapidly acquired chemoresistance^23^. Temozolomide is also recommended in the therapy of LGG because of its significant morbidity and mortality to recurrence and malignant progression^24^. Despite such efforts, the survival rate for patients with GBM and LGG is low, and new and effective treatment methods are still required. In this context, targeting KIFs is a new potential cancer therapy^7^. In the present study, we performed integrative analysis of KIFs in LGG and GBM.

KIFs overexpression disrupts the unique balance of forces associated with normal spindle assembly and function, and thereby leads to the development of spindle defects, genetic instability and tumors^25^. Clinically, we found four clinically significant genes among KIFs: KIF4A, 9, 18A, and 23. Genomic analyses revealed that mutations in the kinesin motor domain of KIF4A and KIF23 resulted in abnormalities of KIFs function, but the percent of upregulated mRNA expression was more prominent than the presence of other specific gene alterations, including amplification, deletions, and missense or truncating mutations. We also examined the correlations between gene alterations and overall survival in LGG and GBM patients, finding that those harboring KIF4A, KIF18A, and KIF23 alterations showed a poorer prognosis than those lacking these alterations in LGG. Additionally, we observed that the mRNA levels of KIF4A, KIF9, KIF18A, and KIF23 were elevated in LGG patients with a higher histological grade and astrocytoma histologic type than in those with a lower histological grade and histologic type, including oligoastrocytoma and oligodendroglioma. In survival analysis using Cutoff Finder, the group with high mRNA expression of KIF4A, 9, 18A, and 23 was significantly associated with a poor prognosis in both LGG and GBM patients.

Several KIFs have been shown to be deeply involved in tumorigenesis and carcinogenesis^11^. The loss of KIF4A leads to tumor formation, and aneuploidy can act as a primary trigger of tumorigenesis^26^. KIF4A was also reported to be significantly increased in lung cancer and cervical cancer^8,27^. In this context, high mRNA expression of KIF4A in our study is also associated with poor prognosis in both LGG and GBM. However, some conflicting results have also been reported. In gastric carcinoma, KIF4 was downregulated and the overexpression of KIF4 repressed the proliferation of cancer cells^28^. Therefore, further studies are needed to obtain a deeper understanding of the complex roles of KIF4A in cancer development and progression.

KIF18A, the kinesin-8 motor protein, plays an essential role in regulating the alignment of chromosomes at the midzone during mitosis^15,29^. Kinesin proteins, including KIF18A, are often deregulated in many types of cancers and are thought to play critical roles in cancer progression. In the present investigation, high KIF18A expression was also significantly associated with poor prognosis and the progression of LGG and GBM. In line with this finding, KIF18A was reported to be involved in the progression of many other types of cancer. It was reported that high KIF18A expression is correlated with an unfavorable prognosis and promotes proliferation, invasion, and metastasis by promoting the cell cycle signaling pathway in hepatocellular carcinoma^30,31^. It was also reported that high KIF18A expression is significantly associated with the progression of breast cancer, renal cell carcinoma, and colon cancer^32–34^. However, conflicting results have also been reported that lower expression of KIF18A is associated with poor prognosis of gastric cancer patients^35^.

KIF23 overexpression was significantly associated with high-grade glioma, as well as higher mortality, in survival analysis, and the downregulation of KIF23 was reported to suppress glioma proliferation^36,37^. A high level of KIF23 expression was observed in most malignant pleural mesothelioma cases and is associated with poor survival^38^. High expression of KIF23 was also shown to be closely associated with poor survival in lung adenocarcinoma^39^. However, conflicting results have also been reported, in that hepatocellular carcinoma patients with aberrant expression of KIF23 had longer survival^40^. In our study, the GSEA results suggested that elevated KIF4A, KIF18A, and KIF23 levels in LGG and GBM patients are correlated with E2F transcription factor activity, the G_2_M checkpoint, Myc targets, and the mitotic spindle. E2F-target gene overexpression is correlated with a poor prognosis in cancer patients by promoting chromosome instability^41^.

KIF9 has recently been implicated in maintaining a physical connection between the centrosome and the nucleus. It is anchored to the nucleus and generates a pulling force that reels in the centrosome up against the nucleus^42^. The Ras superfamily member Gem contributes to mitotic progression by maintaining the correct spindle length through KIF9^17^. However, no study on the relationship between KIF9 and cancer has been reported to date. In this study, high KIF9 expression was associated with cancer progression and showed significantly poor survival, especially in GBM patients. Interestingly, KIF4A, 18A, and 23 showed significant association with intracellular localization, cooccurrence, gene networks, and enrichment pathways, but KIF9 showed significant differences independently in the above analyses. Unlike the GSEA results for KIF4A, 18A, and 23, high mRNA expression levels of KIF9, along with significantly lower expression of mitotic spindle, G_2_M, and E2F target genes, were significantly associated with EMT and angiogenesis.

In TCGA data, histologically normal samples dissected adjacent to the tumor are frequently designated as healthy control samples for cancer studies under the assumption that histological normalcy implies biological normalcy^43^. However, little is known about normal adjacent to tumor tissue on the molecular level and whether it is truly “normal”. This may be particularly relevant to the case or Kif9, whose expression appears to correlate with inflammatory signaling. Furthermore, the issue of stromal admixture with tumor is even more relevant in low grade gliomas, which tend to be more infiltrative, and it would be expected that gene expression data from tissue samples of low grade tumors would be a mixture of tumor and normal/reactive brain signatures. However, due to limitations in informatics analysis methods, it is difficult to confirm sufficiently. The topic needs to be investigated experimentally on further study.

In summary, our results suggest that the expression levels of KIF4A, 9, 18A, and 23 are significantly associated with poor overall survival, and their underlying mechanism about not only G2M check point, E2F targets, mitotic spindle and Myc targets but also EMT and angiogenesis was related to a worse prognosis. We suggest that KIF4A, 9, 18A, and 23 are novel biomarkers and could be targets for treating LGG and GBM.

## Materials and Methods

### Analysis of the mRNA and protein expression data

RNAseqV2-RSEM_genes and clinical data from 1,110 low-grade glioma and glioblastoma samples were obtained from Firebrowse for the analysis of gene expression (http://firebrowse.org/) (Table 2). The raw data were initially analyzed using R (v.3.2.5, http://www.r-project.org). cBioportal (http://www.cbioportal.org/)^21,22^ and Firebrowse (http://firebrowse.org) were used to analyze the mRNA expression and gene alterations. All protein expression and immunohistochemical staining data were downloaded from The Human Protein Atlas (HPA) portal (http://www.proteinatlas.org)^18–20^.

**Table 2:** Clinicopathologic information of low grade glioma and glioblastoma patients.

Feature	Total
Number	1110 (100%)
Disease type	1110 (100%)
Low grade glioma	515 (46%)
Glioblastoma	595 (54%)
**Sex**	
Low grade glioma	515 (100%)
Female	230 (45%)
Male	285 (55%)
Glioblastoma	595 (100%)
Female	230 (39%)
Male	365 (61%)
**Age**	
Low grade glioma	515 (100%)
≤43 years	290 (56%)
>43 years	225 (44%)
Glioblastoma	595 (100%)
≤58 years	289 (49%)
>58 years	306 (51%)
**Histologic type**	
Low grade glioma	515 (100%)
Atrocytoma	194 (38%)
Oligoastrocytoma	130 (25%)
Oligodendroglioma	191 (37%)
Glioblastoma	595 (100%)
Treated primary GBM	20 (3.4%)
Untreated primary (de novo) GBM	544 (91%)
Glioblastoma	31 (5.2%)
**Histologic grade**	
Low grade glioma	515 (100%)
G2	249 (48%)
G3	265 (51%)
**Laterality**	
Low grade glioma	515 (100%)
Left	250 (49%)
Right	253 (49%)
Midline	7 (1.4%)
**Tumor location**	
Low grade glioma	515 (100%)
Frontal lobe	302 (59%)
Temporal lobe	146 (28%)
Parietal lobe	47 (9%)
Occipital lobe	8 (2%)
Cerebellum	2 (0.4%)
Brain stem	1 (0.2%)
**Vital status**	
Low grade glioma	515 (100%)
Alive	389 (76%)
Dead	126 (24%)
Glioblastoma	595 (100%)
Alive	102 (17%)
Dead	491 (83%)

### Functional enrichment analysis

Gene Set Enrichment Analysis (GSEA) was utilized to enrich the mRNAs predicted to correlate with pathways in the hallmark and curated C2 gene sets, Reactome database (www.reactome.org) and the Kyoto Encyclopedia of Genes and Genomes (KEGG) database^44,45^. Enrichment maps were visualized using Cytoscape (v.3.5.1, www.cytoscape.org). A p-value < 0.05 was deemed to indicate statistical significance.

### Survival analysis by Cutoff Finder

Cutoff Finder (http://molpath.charite.de/cutoff) was used to determine the cutoff values in LGGGBM mRNA expression using the log-rank test. Illuminahiseq_maseqv2-RSEM_genes_normalised RNA-seq data of KIF genes were uploaded from tab-separated files; the rows represent patients and columns represent variables (http://molpath.charite.de/cutoff/load.jsp). The cutoff value determination in Cutoff Finder was based on survival, with significance based on the log-rank test for patient outcome (http://molpath.charite.de/cutoff/assign.jsp). The cumulative event (death) rate was calculated by the Kaplan–Meier method using the time to the first event as the outcome variable. The survival date in statistical analysis was determined by the date of operation and date of death. Survival curves were compared by the log-rank test for various recurrence factors. A p-value < 0.05 was deemed to indicate statistical significance.

### Statistical analysis

Statistical analyses were performed using Prism software (v.5.0; GraphPad Prism Software, La Jolla, CA, USA) and Statistical Package for the Social Sciences for Windows (SPSS 13.0, Inc., Chicago, IL, USA). Distributions between groups were compared by *t*-test (or the Kolmogorov–Smirnov test when the expected frequency within any cell was <5) for continuous variables and χ^2^ test (or Fisher’s exact test when the expected frequency within any cell was <5) for categorical variables. Distributions of the characteristics among three or more groups were compared by ANOVA. A p-value < 0.05 was deemed to indicate statistical significance.

## Supplementary information

Supplementary figures
