# Supplementary material for: Integrative analysis of KIF4A, 9, 18A, and 23 and their clinical significance in low-grade glioma and glioblastoma
Source: Sci Rep. 2019 Mar 14;9:4599. doi: 10.1038/s41598-018-37622-3 (PMC6418229; doi:10.1038/s41598-018-37622-3)
Supplement: Supplementary file 1 — Supplementary figures [file 41598_2018_37622_MOESM1_ESM.pdf]

Integrative analysis of KIF4A, 9, 18A, and 23 and their clinical significance in low-grade glioma and glioblastoma

Sang Yeon Cho[1,*], Sungha Kim[2,*], Gwanghun Kim[3], Parul Singh[4], Dong Woon Kim[1,5]

[1]*Department of Anatomy, Brain Research Institute, Chungnam National University School of Medicine, Daejeon 35015, Republic of Korea.*

[2]*Department of Clinical Research, Korea Institute of Oriental Medicine, Daejeon 34054, Republic of Korea.*

[3]*Department of Biomedical Sciences, Seoul National University College of Medicine, Seoul 03080, Republic of Korea.*

[4]*Department of Microbiology and Immunology, Chonbuk National University School of Medicine, Jeonju 54907, Republic of Korea.*

[5]*Department of Medical Science, Chungnam National University School of Medicine, Daejeon 35015, Republic of Korea.*

**These authors contributed equally to this work.*

Corresponding authors: Dong Woon Kim, DVM, PhD.

- *Address: Department of Anatomy, Brain Research Institute, Chungnam National University School of Medicine, 266 Munwha-ro, Jung-gu, Daejeon 35015, Republic of Korea*
- *E-mail: visnu528@cnu.ac.kr (D. W. Kim)*

Running title: KIF4A, 9, 18A and 23 in glioma

Supplementary Legends

Supplementary Figure 1

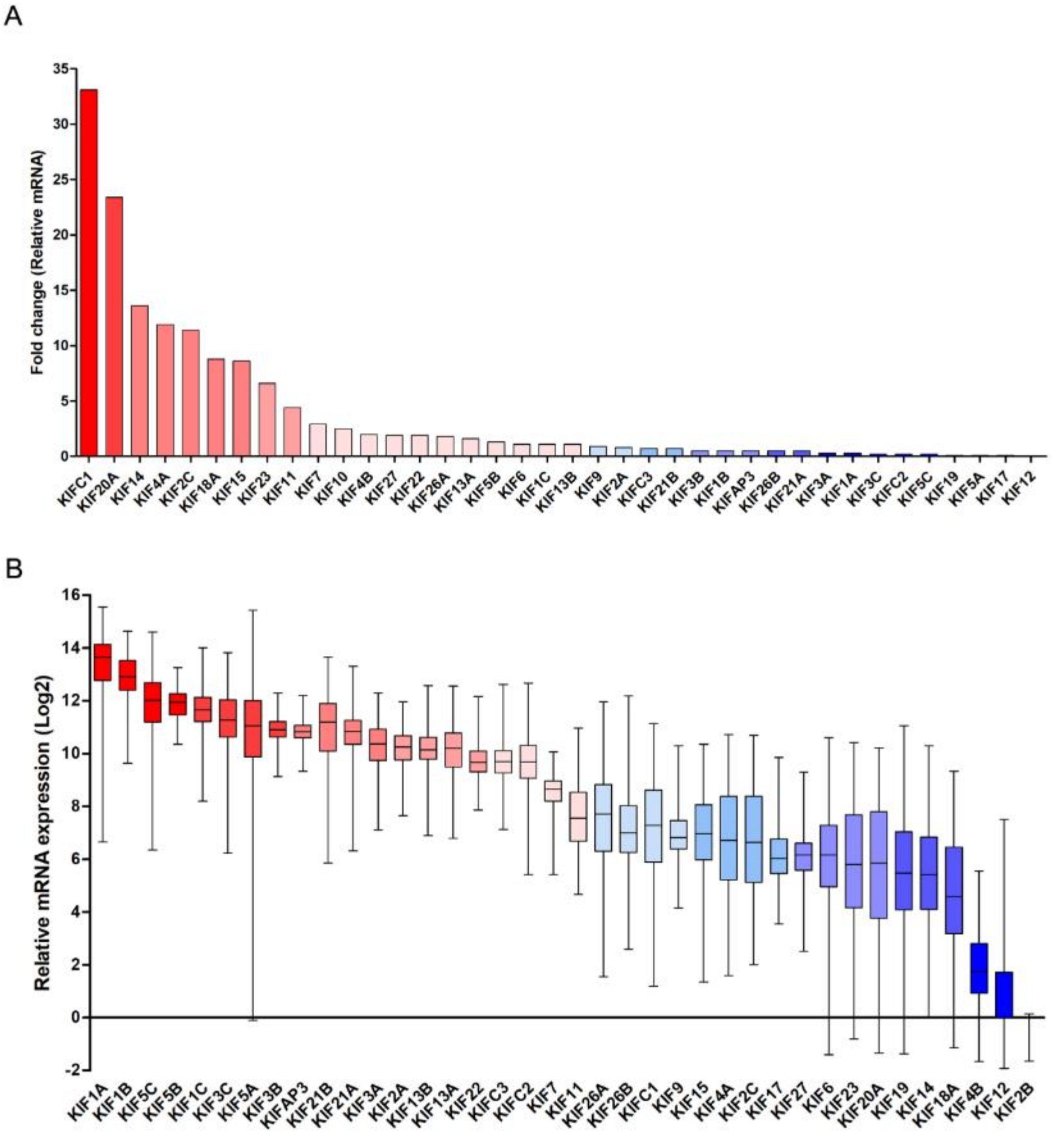

Supplementary Figure 1. mRNA expression of KIF family genes in LGG/GBM. (A) The fold change of mRNA expression of KIF family genes compared with that of normal control samples was examined. (B) The relative mRNA expression of KIF family genes in LGG/GBM was compared.

Supplementary Figure 2

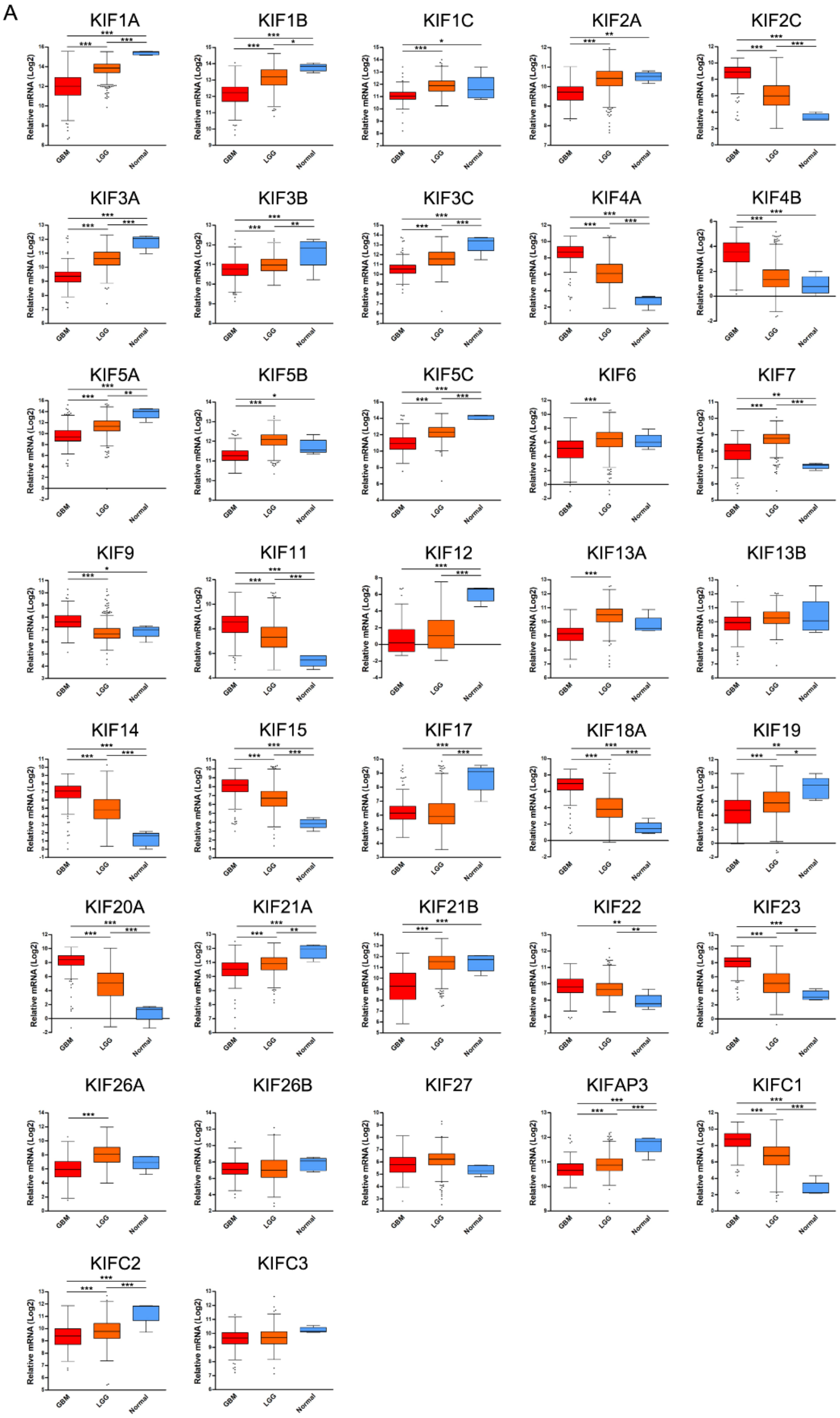

Supplementary Figure 2. (A) The mRNA expression of KIF family genes among normal control samples, LGG, and GBM was examined.

Supplementary Figure 3

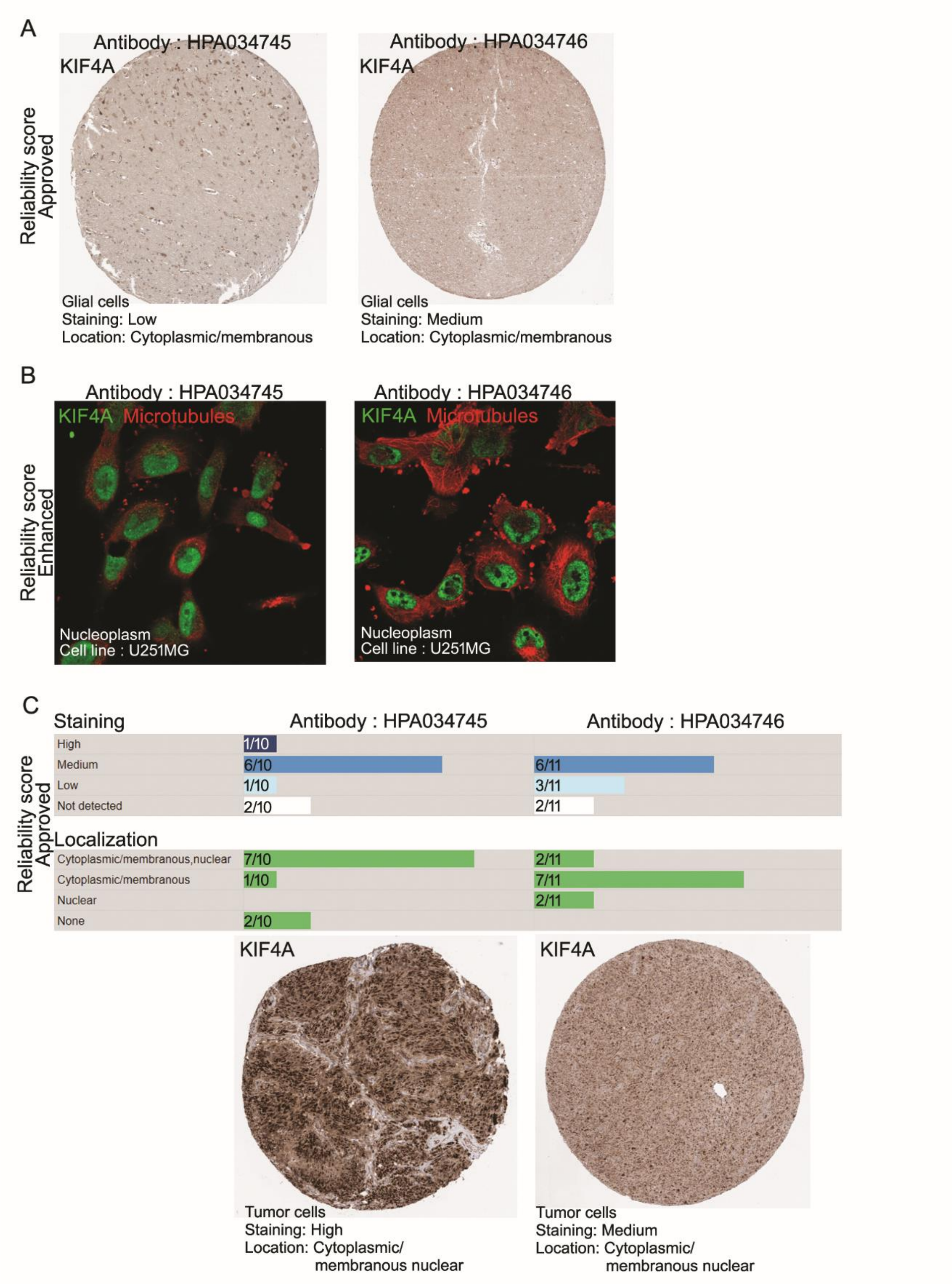

Supplementary Figure 3. Protein expression pattern of KIF4A were examined in The Human Protein Atlas (HPA) portal (http://www.proteinatlas.org)[16-18]. (A) Glial cells of

normal cerebral cortex. (B) Glioblastoma cell lines (U251MG). (C) Glioma tissues. In HPA, all images of tissues stained by immunohistochemistry are manually annotated by a specialist followed by verification by a second specialist. The Reliability score leads to the assignment into one of the following four classes: enhanced, supported, approved and uncertain. Two antibodies for KIF4A were utilized in protein staining (HPA034745 and HPA034746). Image/data available from v18.proteinatlas.org (https://www.proteinatlas.org/ENSG00000090889-KIF4A/antibody). the Human Protein Atlas is licensed under a CC BY-SA license (https://creativecommons.org/licenses/by-sa/4.0/deed.en).

Supplementary Figure 4.

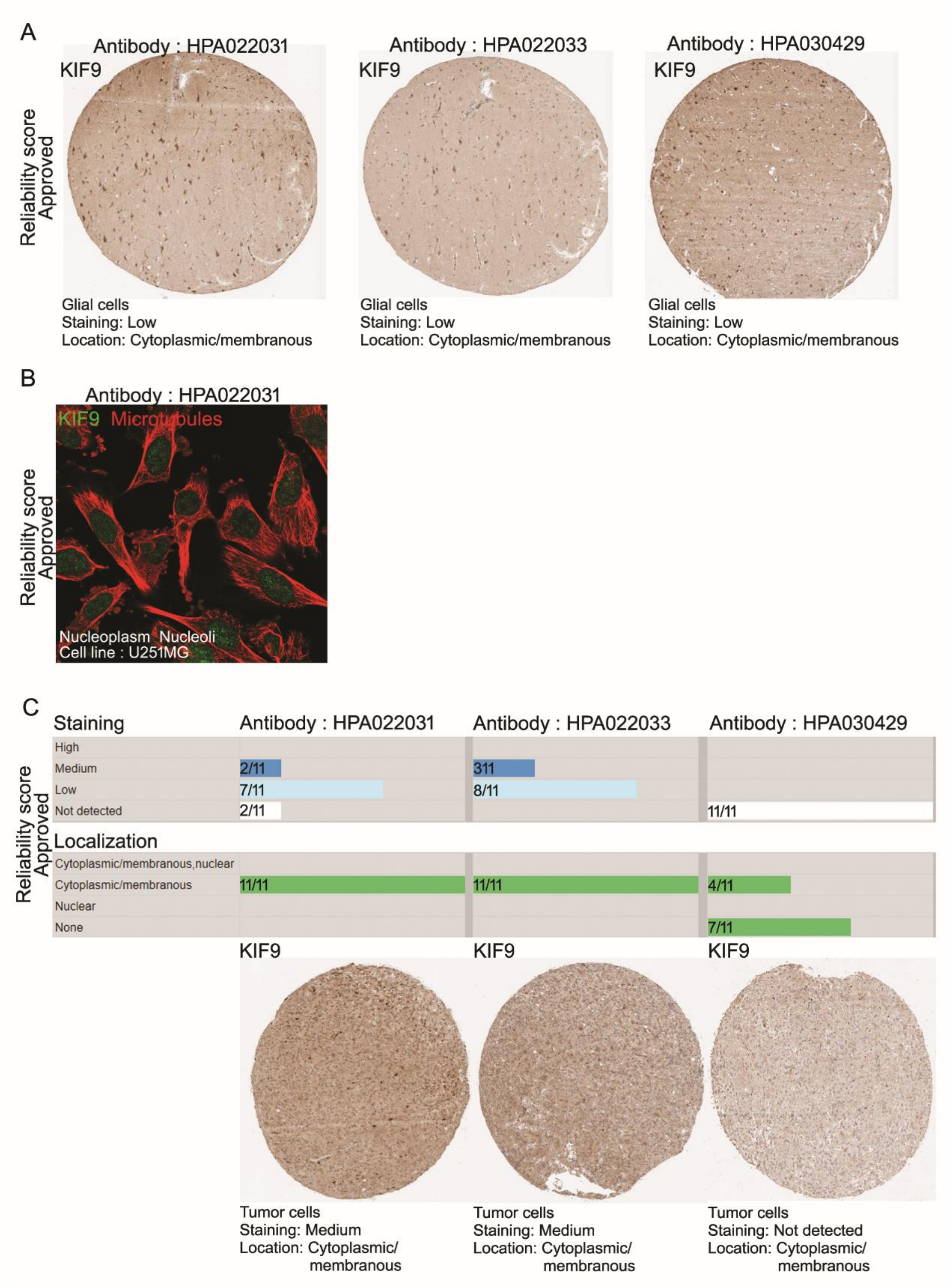

Supplementary Figure 4. Protein expression pattern of KIF9 were examined in The Human Protein Atlas (HPA) portal (http://www.proteinatlas.org)[16-18]. (A) Glial cells of

normal cerebral cortex. (B) Glioblastoma cell lines (U251MG). (C) Glioma tosses. In HPA, all images of tissues stained by immunohistochemistry are manually annotated by a specialist followed by verification by a second specialist. The Reliability score leads to the assignment into one of the following four classes: enhanced, supported, approved and uncertain. Three antibodies for KIF9 were utilized in protein staining (HPA022031, HPA022033 and HPA030429). Image/data available from v18.proteinatlas.org (https://www.proteinatlas.org/ENSG00000088727-KIF9/antibody). the Human Protein Atlas is licensed under a CC BY-SA license (https://creativecommons.org/licenses/by-sa/4.0/deed.en).

Supplementary Figure 5.

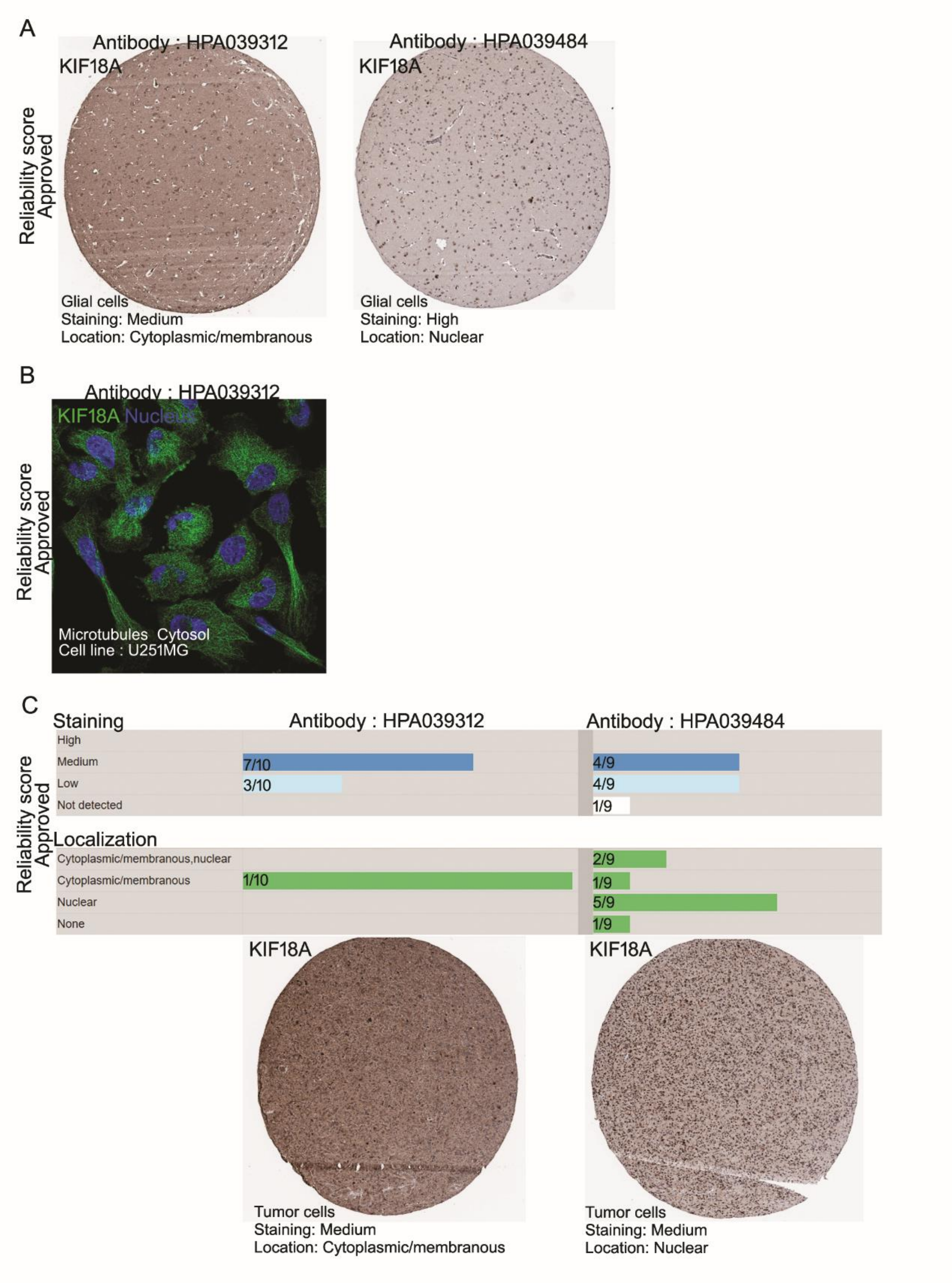

Supplementary Figure 5. Protein expression pattern of KIF18A were examined in The Human Protein Atlas (HPA) portal (http://www.proteinatlas.org)[16-18]. (A) Glial cells of normal cerebral cortex. (B) Glioblastoma cell lines (U251MG). (C) Glioma tissues. In

HPA, all images of tissues stained by immunohistochemistry are manually annotated by a specialist followed by verification by a second specialist. The Reliability score leads to the assignment into one of the following four classes: enhanced, supported, approved and uncertain. Two antibodies for KIF18A were utilized in protein staining (HPA039312 and HPA039484). Image/data available from v18.proteinatlas.org (https://www.proteinatlas.org/ENSG00000121621-KIF18A/antibody). the Human Protein Atlas is licensed under a CC BY-SA license (https://creativecommons.org/licenses/by-sa/4.0/deed.en).

Supplementary Figure 6

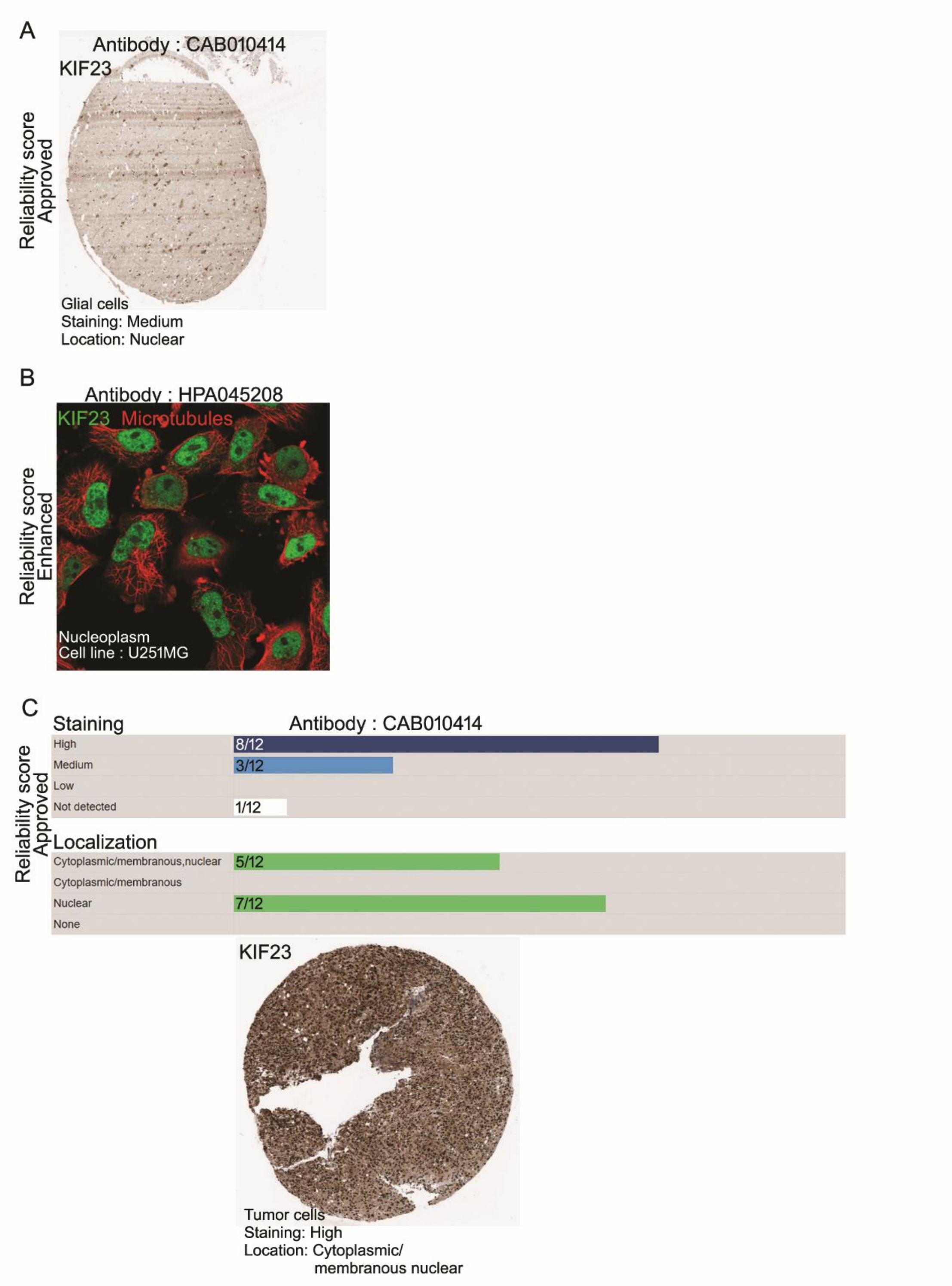

Supplementary Figure 6. Protein expression pattern of KIF23 were examined in The Human Protein Atlas (HPA) portal (http://www.proteinatlas.org)[16-18]. (A) Glial cells of normal cerebral cortex. (B) Glioblastoma cell lines (U251MG). (C) Glioma tissues. In

HPA, all images of tissues stained by immunohistochemistry are manually annotated by a specialist followed by verification by a second specialist. The Reliability score leads to the assignment into one of the following four classes: enhanced, supported, approved and uncertain. Two antibodies for KIF23 were utilized in protein staining (HPA045208 and CAB010414). Image/data available from v18.proteinatlas.org (https://www.proteinatlas.org/ENSG00000137807-KIF23/antibody). the Human Protein Atlas is licensed under a CC BY-SA license (https://creativecommons.org/licenses/by-sa/4.0/deed.en).

Supplementary Figure 7

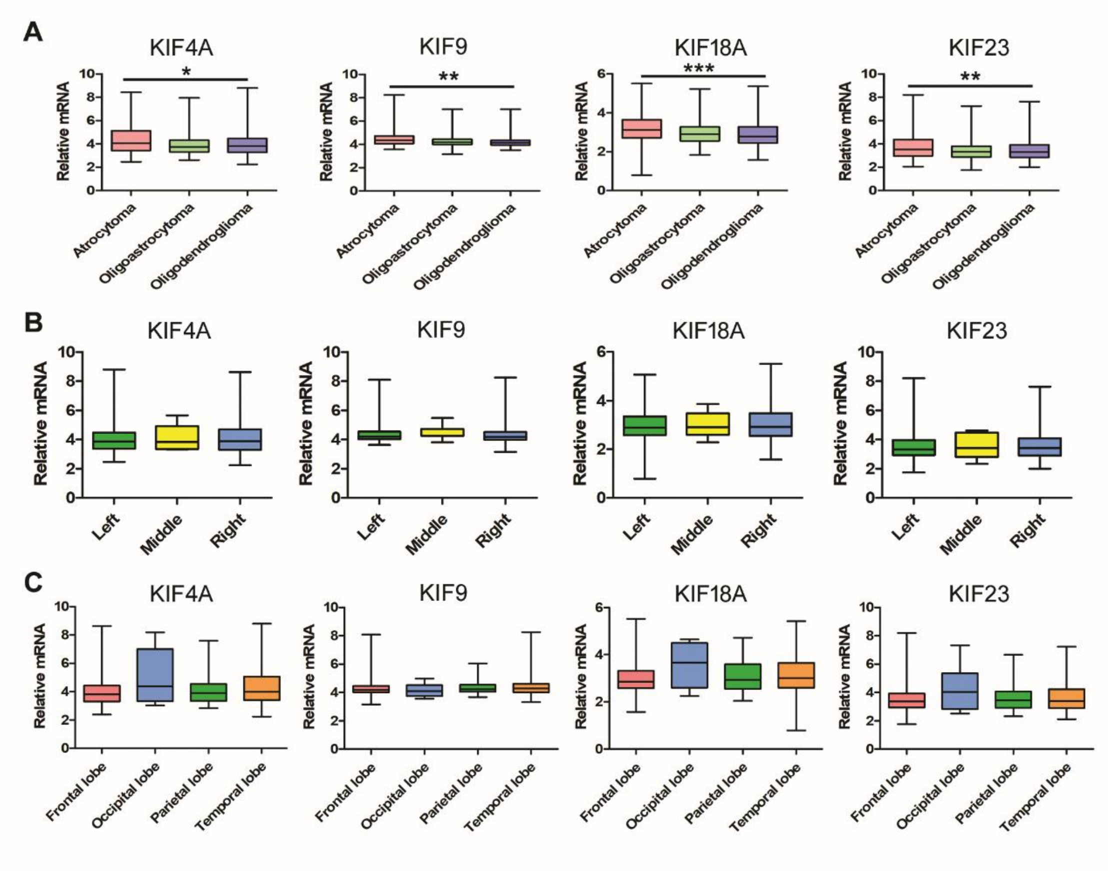

Supplementary Figure 7. (A) Comparison of the mRNA expression of KIF4A, 9, 18A, and 23 in LGG between histologic types, including astrocytoma, oligoastrocytoma, and oligodendroglioma. The distribution was compared between two groups by *t*-test. A p-value <0.05 was deemed to indicate statistical significance. (B) mRNA expression of KIF4A, 9, 18A, and 23 in LGG according to laterality, including left, middle, and right. (C) mRNA expression of KIF4A, 9, 18A, and 23 in LGG according to brain lobe, including frontal lobe, occipital lobe, parietal lobe, and temporal lobe. Statistical analysis was based on one-way ANOVA. A p-value <0.05 was deemed to indicate statistical significance. *p < 0.05, **p < 0.01, ***p < 0.001

Supplementary Figure 8.

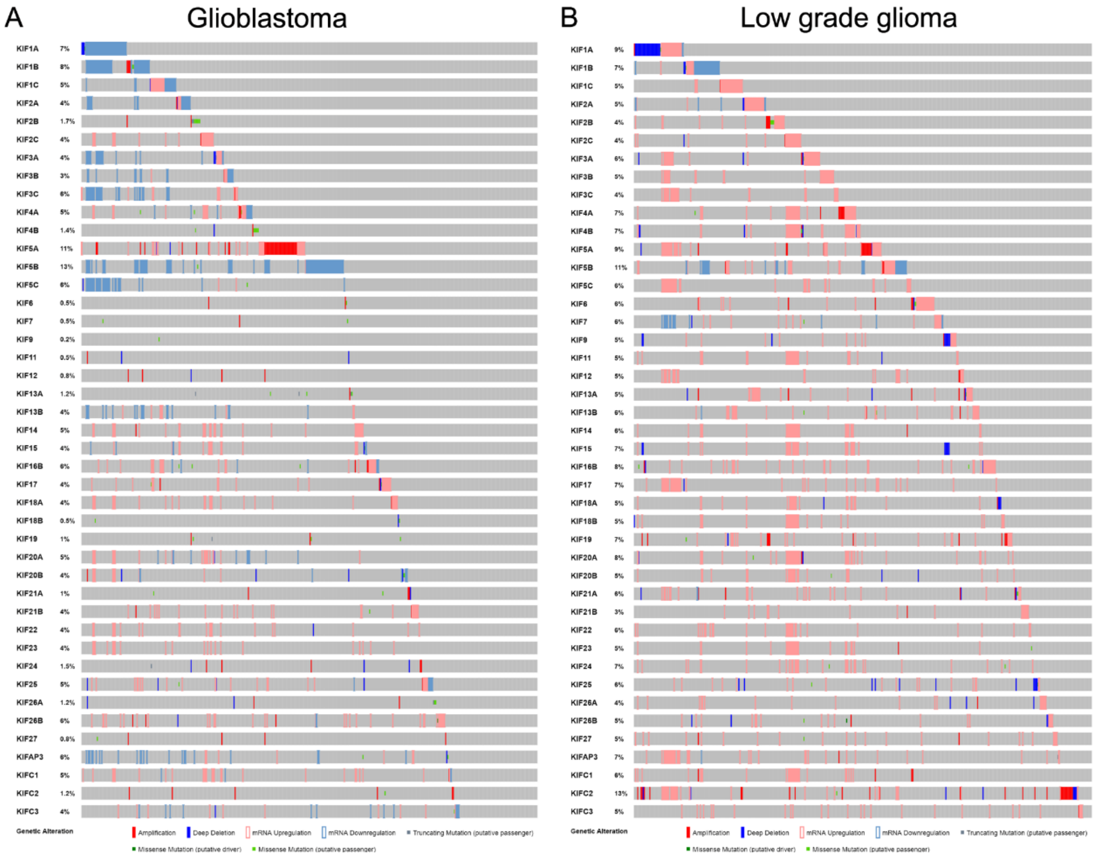

Supplementary Figure 8. (A) Oncoprint of KIF family genes in GBM. (B) Oncoprint of KIF family genes in LGG.

Supplementary Figure 9

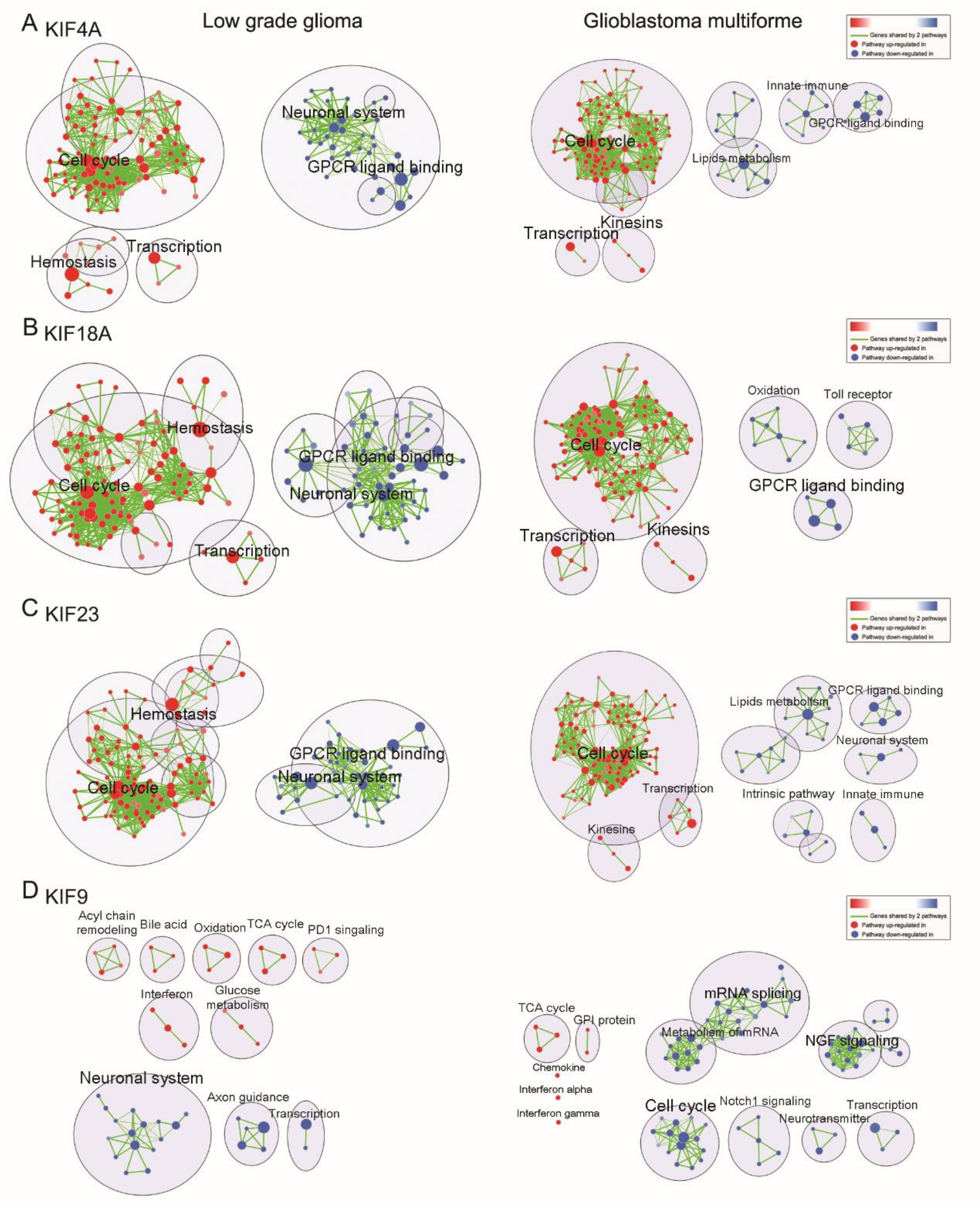

Supplementary Figure 9. The map displays the enriched gene sets based on pathways provided by the Reactome pathway database between the top 10% and bottom 10% of groups of KIF4A, 18A, 23, and 9 (A–D). The red node color represents enrichment in the top 10% group of the gene, where blue represents enrichment in the bottom 10% group of the gene. The color intensity is proportional to enrichment significance.

Supplementary Figure 10

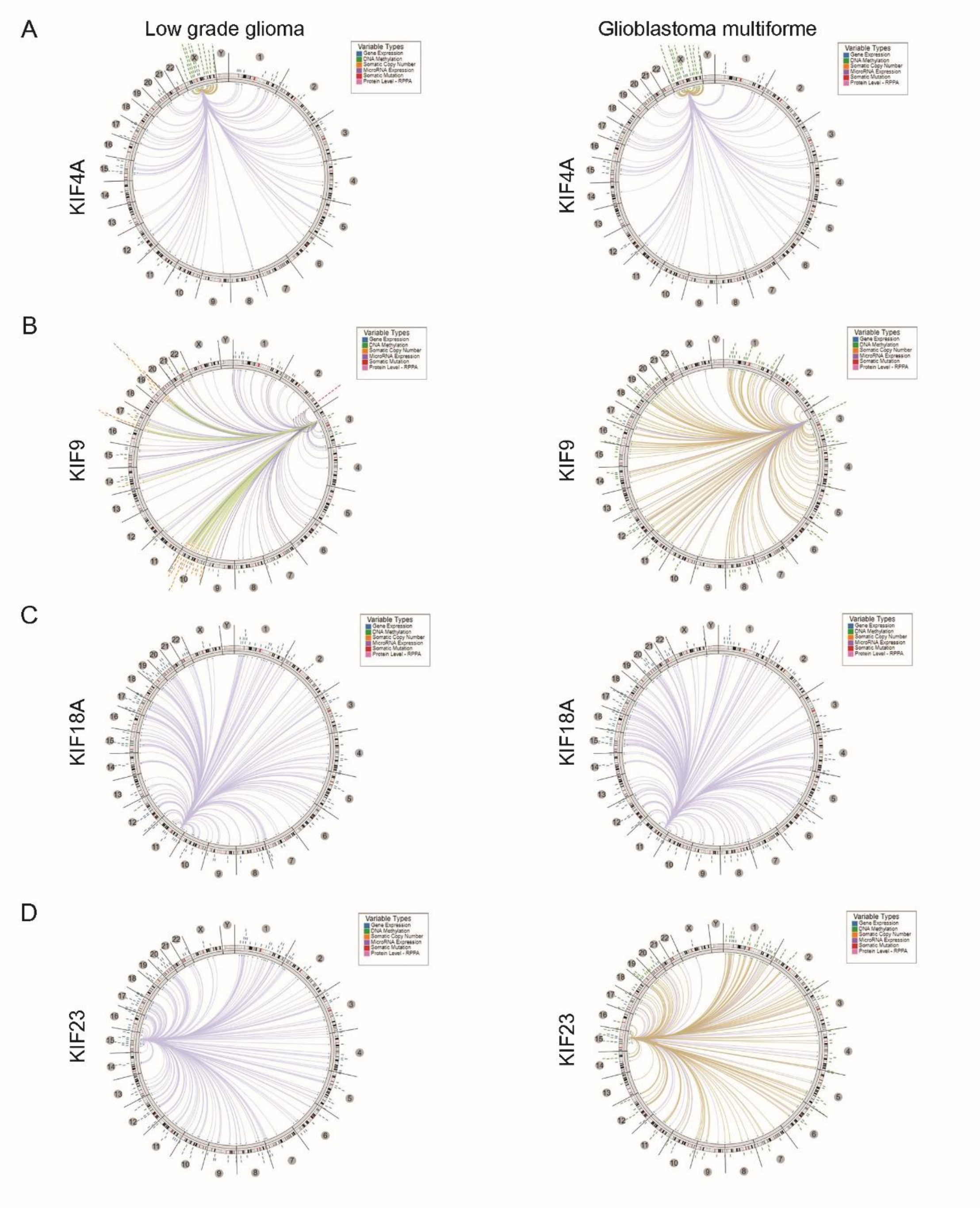

Supplementary Figure 10. Circular plot analysis of KIF4A, 9, 18A, and 23 was performed using The Regulome Explorer and TCGA database based on mutations, methylation, and gene expression. Circular plot of the genome shows the associations and features color lines between genes with gene expression level changes and mutations in LGG and GBM. (A) The association between KIF4A and other genes is

depicted as colored lines at sites of the 23 chromosomes. (B) The correlation between KIF9 and other genes is illustrated as colored lines at sites of the 23 chromosomes. (C) The connection between KIF18A and other genes is shown by color lines on 23 chromosomes. (D) The relationship between KIF23 and other genes is represented by colored lines at sites on the 23 chromosomes.

Supplementary Figure 11

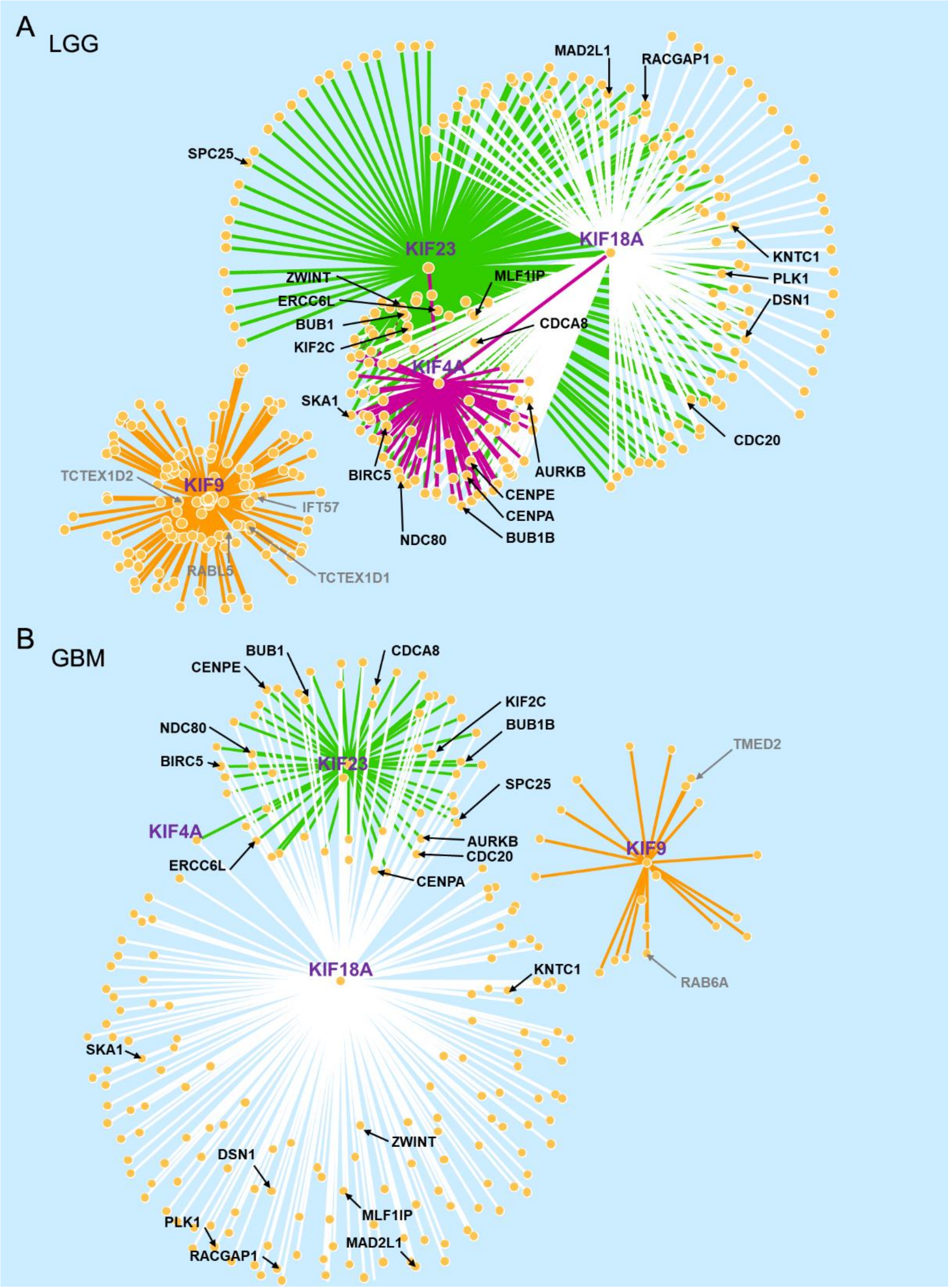

Supplementary Figure 11. (A) Networks of KIF4A, 9, 18A, 23, and their associated genes were depicted and analyzed by Reactome database (www.reactome.org) in LGG. (B) Networks of KIF4A 9, 18A, 23, and their associated genes were depicted and analyzed by Reactome database (www.reactome.org) in GBM.
